# Isolation and Characterization of Two Lytic Bacteriophages, φSt2 and φGrn1; Phage Therapy Application for Biological Control of *Vibrio alginolyticus* in Aquaculture Live Feeds

**DOI:** 10.1371/journal.pone.0151101

**Published:** 2016-03-07

**Authors:** Panos G. Kalatzis, Roberto Bastías, Constantina Kokkari, Pantelis Katharios

**Affiliations:** 1 Institute of Marine Biology, Biotechnology and Aquaculture, Hellenic Centre for Marine Research, Former American Base of Gournes, Heraklion 71003, Crete, Greece; 2 Marine Biological Section, University of Copenhagen, Helsingør, Denmark; 3 Institute of Biology, Pontificia Universidad Católica de Valparaíso, Valparaíso, Chile; University of Aveiro, PORTUGAL

## Abstract

Bacterial infections are a serious problem in aquaculture since they can result in massive mortalities in farmed fish and invertebrates. Vibriosis is one of the most common diseases in marine aquaculture hatcheries and its causative agents are bacteria of the genus *Vibrio* mostly entering larval rearing water through live feeds, such as *Artemia* and rotifers. The pathogenic *Vibrio alginolyticus* strain V1, isolated during a vibriosis outbreak in cultured seabream, *Sparus aurata*, was used as host to isolate and characterize the two novel bacteriophages *φ*St2 and *φ*Grn1 for phage therapy application. *In vitro* cell lysis experiments were performed against the bacterial host *V*. *alginolyticus* strain V1 but also against 12 presumptive *Vibrio* strains originating from live prey *Artemia salina* cultures indicating the strong lytic efficacy of the 2 phages. *In vivo* administration of the phage cocktail, *φ*St2 and *φ*Grn1, at MOI = 100 directly on live prey *A*. *salina* cultures, led to a 93% decrease of presumptive *Vibrio* population after 4 h of treatment. Current study suggests that administration of *φ*St2 and *φ*Grn1 to live preys could selectively reduce *Vibrio* load in fish hatcheries. Innovative and environmental friendly solutions against bacterial diseases are more than necessary and phage therapy is one of them.

## Introduction

The intensification of aquaculture production has dramatically increased the incidences of microbial diseases causing substantial economic losses to the industry. One of the biggest problems in intensive fish culture is the mass mortalities in fish larvae caused by bacterial infections [1–3]. In marine aquaculture, vibrios are major pathogens causing vibriosis which is the most common disease in marine fish and invertebrate hatcheries [3–7].

*Vibrio alginolyticus* is a ubiquitous bacterium found in marine environment that has been associated with disease in aquatic animals but also in humans, causing tissue damages in skin, ear and internal organs [8–11].

*V*. *alginolyticus* is also one of the most common species found in marine hatchery water [12,13] and it is considered as an important pathogen for marine organisms [14], especially by being opportunistic invader of already damaged fish tissues [15]. There are several reports for *V*. *alginolyticus* causing significant mortalities in cultured gilthead seabream, *S*. *aurata*, especially during early life stages [16–20]. Larval enteropathy (LE) is the most important pathology affecting this species at hatcheries, which is responsible for great reduction in survival rates. *V*. *alginolyticus* alone, or in synergy with other bacteria such as *Aeromonas hydrophila*, constitutes the major causative agent of LE [19]. Apart from cultured gilthead seabream, *V*. *alginolyticus* infection has been recorded during early rearing stages (≤3 g) of sharpsnout seabream, *Diplodus puntazzo* [21]. Mortality due to *V*. *alginolyticus* has also been recorded in ornamental fish [22–24] and several invertebrates such as *Penaeus monodon* [25] and *Macrobrachium rosenbergii* [26]. In aquaculture there is a general consensus that *V*. *alginolyticus* enters the system through live prey (artemia and rotifers) which serve as vehicles for introducing the bacteria into the hatchery tanks [27–29]. There are several studies demonstrating that *Artemia* nauplii are vectors for potentially harmful bacteria such as *Vibrio* spp. [30]. *V*. *alginolyticus* has been reported as the dominant member of the cultivable bacterial community of *Artemia* [13,29,31,32].

Disinfection techniques (filters, ozone, UV etc.) in marine hatcheries cannot offer a completely bacteria—free environment [33] and may lead to microbial imbalance leaving environmental niche wide open for the proliferation of opportunistic pathogens [34,35]. Administration of antibiotics has traditionally been the most commonly applied strategy against bacterial infections. Today, antibiotic usage is becoming increasingly obsolete in aquaculture as many economically important pathogens evolve resistance, including strains belonging to the genera *Aeromonas* and *Vibrio* [18,36–38]. Development of multi—drug resistant strains, disturbance of natural microbiota, ecological and public health issues are some of the most important problems caused by the excessive use of chemotherapy [39–41]. Thus, bacterial disease outbreaks could be ideally managed by limiting or even excluding pathogenic bacteria, as *Vibrio*, from the system without affecting the beneficial microbes. In fragile systems like marine hatcheries the use of bacteriophages, viruses that infect bacteria, is a promising alternative since they can selectively remove their bacterial hosts, while leaving natural microbiota unaffected [41,42]. Up to date, phage therapy has been applied in numerous cases of vibriosis against many pathogenic *Vibrio* spp. such as *V*. *harveyi*, *V*. *parahaemolyticus* and *V*. *anguillarum*, with positive results [43–48].

The objective of this study is the isolation and characterization of lytic bacteriophages against the dominant vibrios of the live feeds of a marine fish hatchery and the assessment of their efficacy to reduce vibrio load in live feeds prior to their administration to fish larvae.

## Materials and Methods

### Bacterial strains and growth conditions

*Vibrio alginolyticus*, strain V1 was used as host for bacteriophage isolation. This strain was isolated previously from a vibriosis episode in juvenile gilthead seabream, *S*. *aurata* and it has already been fully sequenced [49]. Twenty-five different bacterial strains belonging to seven *Vibrio* species (*V*. *anguillarum*, *V*. *harveyi*, *V*. *alginolyticus*, *V*. *ordalii*, *V*. *parahaemolyticus*, *V*. *splendidus* and *V*. *owensii*) were used in the current study (Table 1). These bacteria were either purchased from international collections, or belong to HCMR’s collection and include both clinical and environmental isolates. *V*. *splendidus* strains were a kind offer from Dr. Frédérique Le Roux (Roscoff Marine Station). All bacterial species have been identified using biochemical (BIOLOG GEN III) and/or molecular tools [50–52]. All bacterial strains were cultured in artificial sea water (23.4 gL^-1^NaCl, 24.7 gL^-1^ MgSO_4_ x 7H_2_O, 1.5 gL^-1^KCl and 1.43 gL^-1^CaCl_2_ x 2H_2_O), supplemented with 1% tryptone (Difco) and 0.5% yeast extract (Difco) at 25°C with reciprocal shaking [48].

**Table 1:** Bacterial strains of the genus *Vibrio* used in the current study. T: type strain.

#	Strains	*Species*	Locality	Type
**1**	**V1 (host)**	*V*. *alginolyticus*	Greece	Clinical
**2**	**DSMZ 2171**	*V*. *alginolyticus*	Japan	Collection (T)
**3**	**ValgHCMR**	*V*. *alginolyticus*	Greece	Clinical
**4**	**R5**	*V*. *alginolyticus*	Greece	Environmental
**5**	**R6**	*V*. *alginolyticus*	Greece	Environmental
**6**	**E1**	*V*. *alginolyticus*	Greece	Environmental
**7**	**A1**	*V*. *alginolyticus*	Greece	Environmental
**8**	**V2**	*V*. *alginolyticus*	Greece	Clinical
**9**	**DSMZ 19623**	*V*. *harveyi*	USA	Collection
**10**	**VIB391**	*V*. *harveyi*	Thailand	Clinical
**11**	**Vh2**	*V*. *harveyi*	Greece	Clinical
**12**	**Vh4**	*V*. *harveyi*	Greece	Clinical
**13**	**Vh5**	*V*. *harveyi*	Greece	Clinical
**14**	**VhKar**	*V*. *harveyi*	Greece	Clinical
**15**	**VhSerF**	*V*. *harveyi*	Greece	Clinical
**16**	**VhNo22**	*V*. *harveyi*	Greece	Clinical
**17**	**Va5**	*V*. *splendidus*	Greece	Clinical
**18**	**3Z-31**	*V*. *splendidus*	France	Clinical
**19**	**3H2-4**	*V*. *splendidus*	France	Clinical
**20**	**DY05**	*V*. *owensii*	Australia	Collection (T)
**21**	**Vh3**	*V*. *parahaemolyticus*	Greece	Clinical
**22**	**PF4**	*V*. *anguillarum*	Chile	Clinical
**23**	**VaS**	*V*. *anguillarum*	Greece	Clinical
**24**	**ATCC 19264**	*V*. *anguillarum*	Sweden	Collection
**25**	**ATCC 33509**	*V*. *ordalii*	USA	Collection (T)

### Isolation and propagation of bacteriophages

Two novel bacteriophages were isolated from water samples collected from two locations of the north coastline of Crete, Greece (35°20'16.2"N, 25°06'36.6"E and 35°20'05.3"N, 25°16'30.3"E) through standard enrichment methodology [53]. No special permission was required for sampling water. Briefly, samples were supplemented with 1% tryptone (Difco) and 0.5% yeast extract (Difco) and then inoculated with bacterial host, *V*. *alginolyticus* strain V1. Samples were incubated overnight at 25°C with reciprocal shaking, following centrifugation at 6,000 x g for 10 min. Supernatants were filtered (0.22 μm) and 100 μL were plated by standard double-layer agar method and incubated overnight at 25°C to detect and enumerate plaque forming units (pfu). Isolated plaques were picked and purified by re-plating five times to ensure clonal phage stocks. For phage propagation, 50 mL of a bacterial host liquid culture in early exponential phase (~108 cells mL^-1^) was infected at a multiplicity of infection (MOI) of 10 and incubated overnight at 25°C with reciprocal shaking. After centrifugation of the cultures, the supernatants were filtered (0.22μm), tittered and stored at 4°C.

### Host range and efficiency of plating (EOP)

Bacterial lawns of each bacterial strain tested were prepared on Petri dishes of artificial sea water (23.4 gL^-1^NaCl, 24.7 gL^-1^ MgSO_4_ x 7H_2_O, 1.5 gL^-1^KCl and 1.43 gL^-1^CaCl_2_ x 2H_2_O) supplemented with 1% tryptone (Difco) and 0.5% yeast extract (Difco), and 20 μL drops of each phage were added on them, following overnight incubation at 25°C. EOP assay was also performed to obtain a quantitative measure of phage’s lytic activity and to assess possible “lysis from without” phenomenon [54,55]. EOP was determined for each phage—sensitive bacterial strain, by dividing the infectivity of phages vs tested strains to the infectivity of phages vs host strain V1 [56].

### Morphological characterization of bacteriophages

Virion morphology of isolated phages was observed by Transmission Electron Microscopy (TEM). Samples were prepared on collodium copper grids, negatively contrasted with 2% uranyl acetate, and examined using an electron microscope (JEOL JEM2100) at 80 kV and an instrumental magnification of 120,000.

### Viral whole genome extraction and RFLP analysis

Bacteriophages’ genome was extracted using phenol-chloroform protocol [48] and visualized in agarose gel 0.4% at 30 mV compared to a high range genome size ladder (Thermo Scientific GeneRuler High Range DNA Ladder). Restriction Fragment Length Polymorphism analysis (RFLP) was performed using six different restriction enzymes *Hpa*II, *Sau*3AI, *Hinc*II, *Hae*III, *Bg*III and *Bam*HI, according the manufacturer’s instructions (Promega) for digesting phage genome. The genome of the phages was tested for resistance against RNAse A (Qiagen) and DNAse (Qiagen) treatment.

### One step growth curve of the bacteriophages

Bacteriophages were added to 1mL host bacterial culture in early exponential phase, with MOI = 0.01, following incubation at 25°C for 15 min and centrifugation at 6,000 x g for 10 min. Supernatant containing free phages was discarded whereas phages that managed to attach to the bacteria during interaction time were pelleted on the bottom of the tube. Pellet was suspended in 0.5 mL and then transferred in 20 mL fresh liquid medium. This moment was considered as t = 0 and thereafter, 20 μL drops of serial dilutions were placed on Petri dishes containing bacterial lawn of the host, every 10 min for total duration of 80 min. Phage plaques were counted following overnight incubation at 25°C. The experiment was repeated three times for each phage.

### *In vitro* efficacy vs host strain V1

*In vitro* lysis assay was performed in sterile 96-well plates using a TECAN microplate reader (Infinite PRO 200) equipped with temperature control. Briefly, 24 wells were used per each condition. Wells were loaded with 200 μL of freshly prepared culture of the host bacteria. The plate was placed in the reader and incubated at 25°C with orbital shaking. Cultures were infected at 3 different MOIs (1, 10 and 100 in sextuplicates) when the bacterial culture was at the exponential phase. Twelve wells were not infected and served as control. The growth curve of the cultures was monitored in real—time over 1000 min and OD_560_ measurements were recorded every 20 min.

### *In vitro* efficacy vs presumptive *vibrios* from *A*. *salina* culture

The lytic effect of phages *φ*Grn1 and *φ*St2 was also tested *in vitro* against bacterial isolates from live feed *A*. *salina* culture, grown on the selective medium for *Vibrio* TCBS (Thiosulphate Citrate Bile Salt). A sample from HCMR’s *Artemia* live feed culture was serially diluted on TCBS and incubated at 25°C for 24 h. Twelve different single bacterial colonies covering the whole range of various morphologies were picked and recultured twice on artificial sea water (23.4 gL^-1^NaCl, 24.7 gL^-1^ MgSO_4_ x 7H_2_O, 1.5 gL^-1^KCl and 1.43 gL^-1^CaCl_2_ x 2H_2_O), supplemented with 1% tryptone (Difco) and 0.5% yeast extract (Difco). Bacterial cultures of isolated presumptive vibrios were infected in their early exponential phase by the mixture of *φ*St2 and *φ*Grn1 using a MOI = 100, with both phages’ titer being approximately the same. The experiment was done as described previously for the host strain in a 96 –well plate for 850 min organized in triplicates per bacterium.

### *In vivo* administration of *φ*Grn1 and *φ*St2 in *A*. *salina* culture

The brine shrimp, *Artemia* is a zooplanktonic organism widely used as live feed. It can be hatched within 24 hours from dormant cysts (batch culture) which can be easily distributed and stored for prolonged periods of time [30]. The cultures of *Artemia salina* (SEP Art, INVE), were obtained from the department of live feed cultures of the Hellenic Centre for Marine Research.

The presumptive *Vibrio* load of the *Artemia* cultures had been estimated earlier to be approximately 10^5^ cfu mL^-1^. Six plastic containers were used, each containing 1L of *A*. *salina* cultures supplied with intense aeration. The total presumptive *Vibrio* count in each container was assessed at t_0_ = 0 h and at t_1_ = 4 h following serial dilutions of 1 mL samples and plating in TCBS. Phage mixture of *φ*Grn1 and *φ*St2 was directly administered at a MOI = 100. The experiment was done in triplicates, phage treated vs untreated controls *Artemia* cultures.

### Statistical analysis

Statistical analysis was performed to assess statistical significance of the difference between the two groups (control and phage treated) of the *in vivo* experiment. A two—way ANOVA (factor A: time, factor B: treatment, dependent variable: *Vibrio* population) was performed following assessment of normality of the data distribution. All statistical analyses were performed using SigmaPlot, version 13.0, Systat Software, Inc., San Jose, California, USA, www.sigmaplot.com.

## Results

### Isolation and characterization of bacteriophages *φ*St2 and *φ*Grn1

Enrichment method was used to process 20 water samples. Two of the filtrates producing zones of clearing were selected for purification and further characterization. Bacteriophages *φ*St2 and *φ*Grn1 were successfully isolated and purified after five—times plaque re-plating. The presence of phages was confirmed by single negative colonies formation on the bacterial lawn.

**Fig 1:**
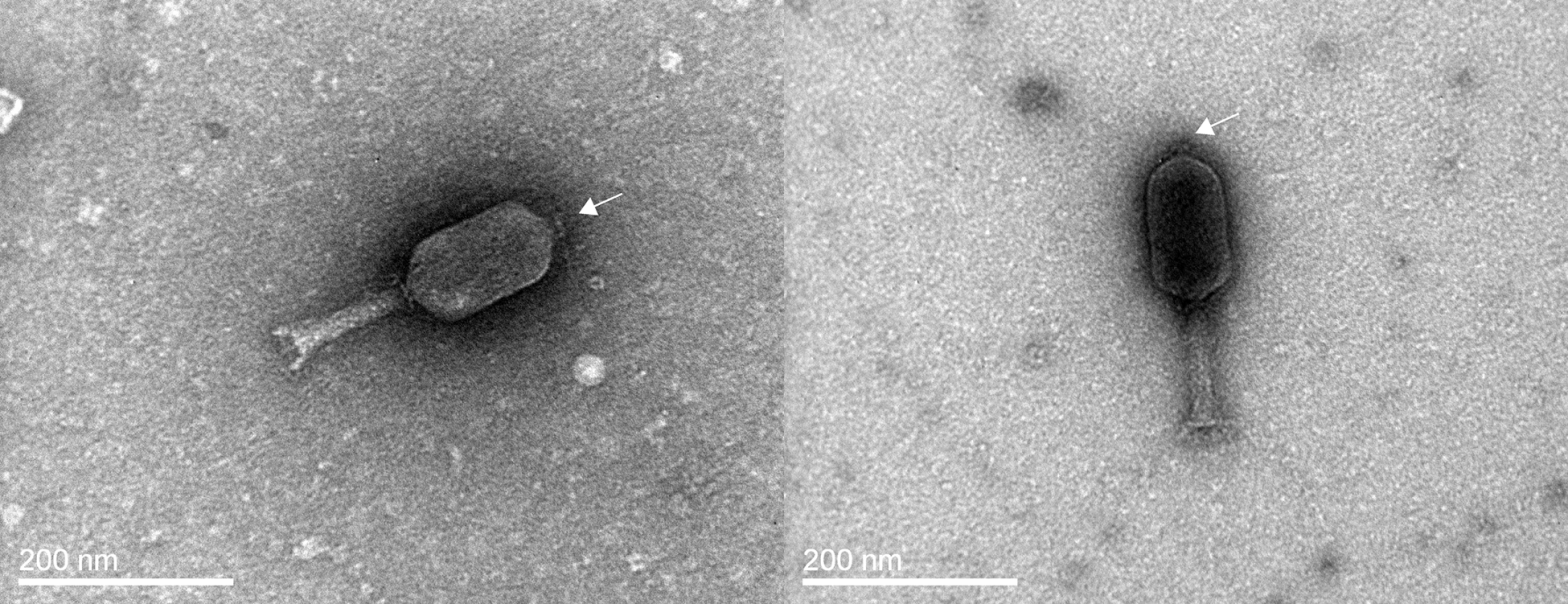
Transmission Electron Microscopy micrographs of *φ*St2 (left) and *φ*Grn1 (right). Both phages have similar morphology and are classified at the *Myoviridae* family. White arrows indicated the filamentous appendage which is present on both phages’ heads.

The morphology of the virions of *φ*St2 and *φ*Grn1 under TEM (Fig 1) classified them to Myoviridae family. Both *φ*Grn1 and *φ*St2 have elongated head and contractile tail. The head of bacteriophage *φ*St2 was approximately 81 nm wide by 151 nm long and the tail was about 132 nm long with a diameter of 20 nm. In the case of bacteriophage *φ*Grn1, the head was approximately 74 nm wide by 138 nm long and the tail was about 134 nm long with a diameter of 20 nm. Furthermore, both phages possessed a filamentous appendage on the top of the head as observed under TEM examination (Fig 1). Both phages produced similar pinhead’s size clear plaques on the host bacterial lawn.

Twenty-five bacterial strains were used to define the lytic spectrum of the novel phages *φ*St2 and *φ*Grn1. Both phages presented the same host range infecting different strains of *V*. *alginolyticus*, one strain of *V*. *harveyi* and one of *V*. *parahaemolyticus*. On the contrary, none of them was able to infect any *V*. *anguillarum*, *V*. *ordalii*, *V*. *owensii* and *V*. *splendidus* strains (Table 2). The lytic activity of phages *φ*St2 and *φ*Grn1 is evident in 40% (10 out of 25) of the bacterial strains tested.

**Table 2:** Host range of phages *φ*St2 and *φ*Grn1 against 25 bacterial strains from 7 different *Vibrio* species: *V*. *anguillarum*, *V*. *ordalii*, *V*. *harveyi*, *V*. *alginolyticus*, *V*. *parahaemolyticus*, *V*. *splendidus* and *V*. *owensii*. Dark grey colour indicates clear lysis, light grey colour indicates turbid lysis and white colour indicates no inhibition. EOP is expressed as: the fraction of phages’ infectivity vs tested strains to phages’ infectivity vs host strain.

Strains	Species	*φ*St2	*φ*Grn1
**V1 (host)**	*V*. *alginolyticus*	1,E+00	1,E+00
**DSMZ 2171**	*V*. *alginolyticus*	6,E-01	9,E-01
**ValgHCMR**	*V*. *alginolyticus*	1,E-05	4,E-05
**R5**	*V*. *alginolyticus*	8,E-01	8,E-01
**R6**	*V*. *alginolyticus*	5,E-05	4,E-04
**E1**	*V*. *alginolyticus*	6,E-01	9,E-01
**A1**	*V*. *alginolyticus*	2,E+00	1,E+00
**V2**	*V*. *alginolyticus*	8,E-01	4,E-01
**DSMZ 19623**	*V*. *harveyi*	0	0
**VIB391**	*V*. *harveyi*	1,E-01	7,E-01
**Vh2**	*V*. *harveyi*	0	0
**Vh4**	*V*. *harveyi*	0	0
**Vh5**	*V*. *harveyi*	0	0
**VhKar**	*V*. *harveyi*	0	0
**VhSerF**	*V*. *harveyi*	0	0
**VhNo22**	*V*. *harveyi*	0	0
**Va5**	*V*. *splendidus*	0	0
**3Z-31**	*V*. *splendidus*	0	0
**3H2-4**	*V*. *splendidus*	0	0
**DY05**	*V*. *owensii*	0	0
**Vh3**	*V*. *parahaemolyticus*	9,E-02	1,E-02
**PF4**	*V*. *anguillarum*	0	0
**VaS**	*V*. *anguillarum*	0	0
**ATCC 19264**	*V*. *anguillarum*	0	0
**ATCC 33509**	*V*. *ordalii*	0	0

**Fig 2:**
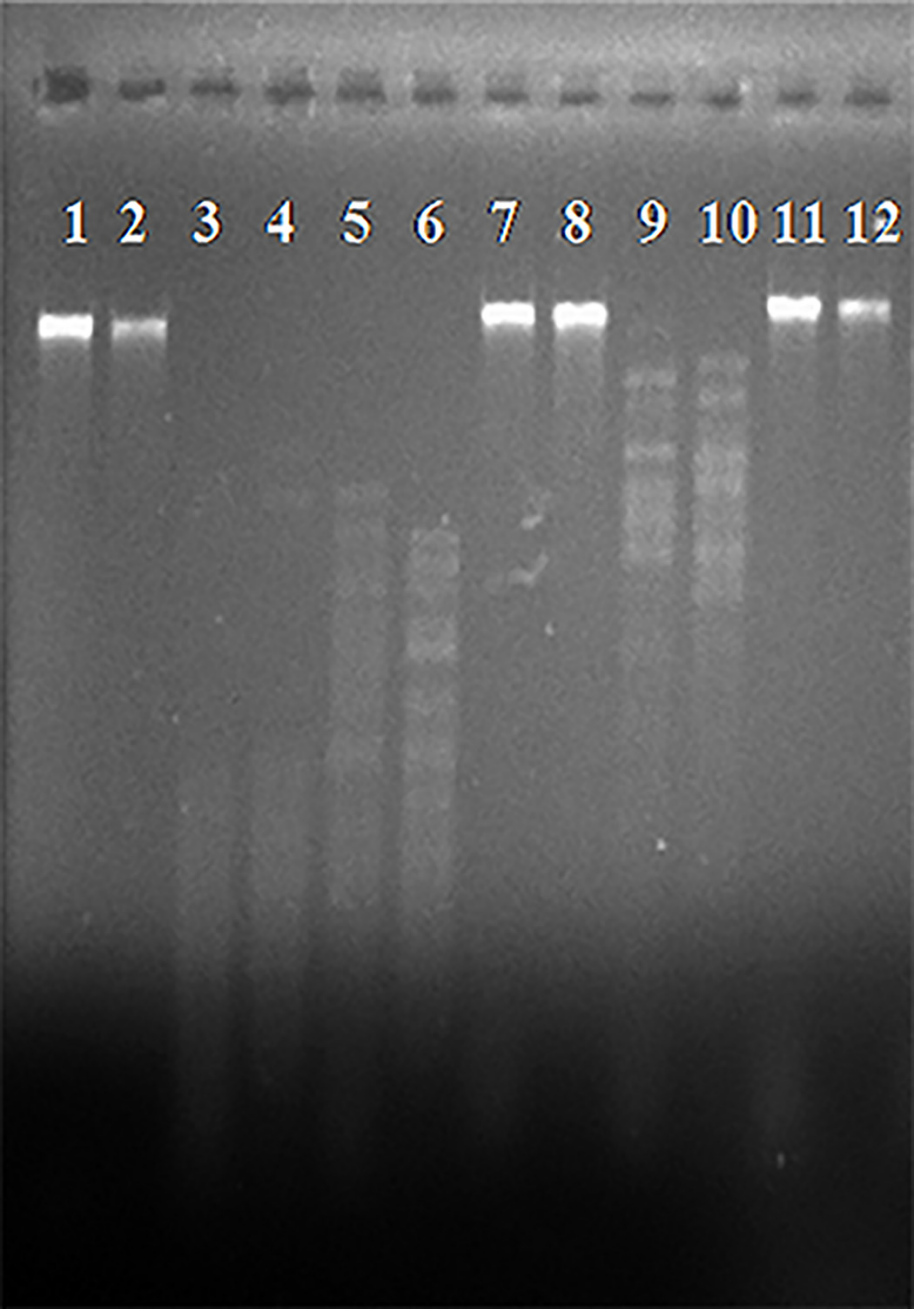
Restriction endonuclease digestion profile of *φ*St2 and *φ*Grn1 DNAs. 1: *φ*St2—*Hpa*II, 2: *φ*Grn1—*Hpa*II, 3: *φ*St2—*Sau*3AI, 4: *φ*Grn1—*Sau*3AI, 5: *φ*St2—*Hinc*II, 6: *φ*Grn1—*Hinc*II, 7: *φ*St2—*Hae*III, 8: *φ*Grn1—*Hae*III, 9: *φ*St2—*Bg*III, 10: *φ*Grn1—*Bg*III, 11: *φ*St2—*Bam*HI, 12: *φ*Grn1—*Bam*HI.

Both bacteriophages contain a double stranded DNA sensitive to DNAse and resistant to RNAse A treatment. *Hpa*II, *Hae*III and *Bam*HI did not digest the genomes of the phages (Fig 2). However, both phages’ genomes were digested by *Sau*3AI, *Hinc*II and *Bg*III indicating the genetic differentiation of these phages (Fig 2). According to unpublished results about the sequences of *φ*St2 and *φ*Grn1 their genome sizes are 250,485 bp and 248,605 bp, respectively.

**Fig 3:**
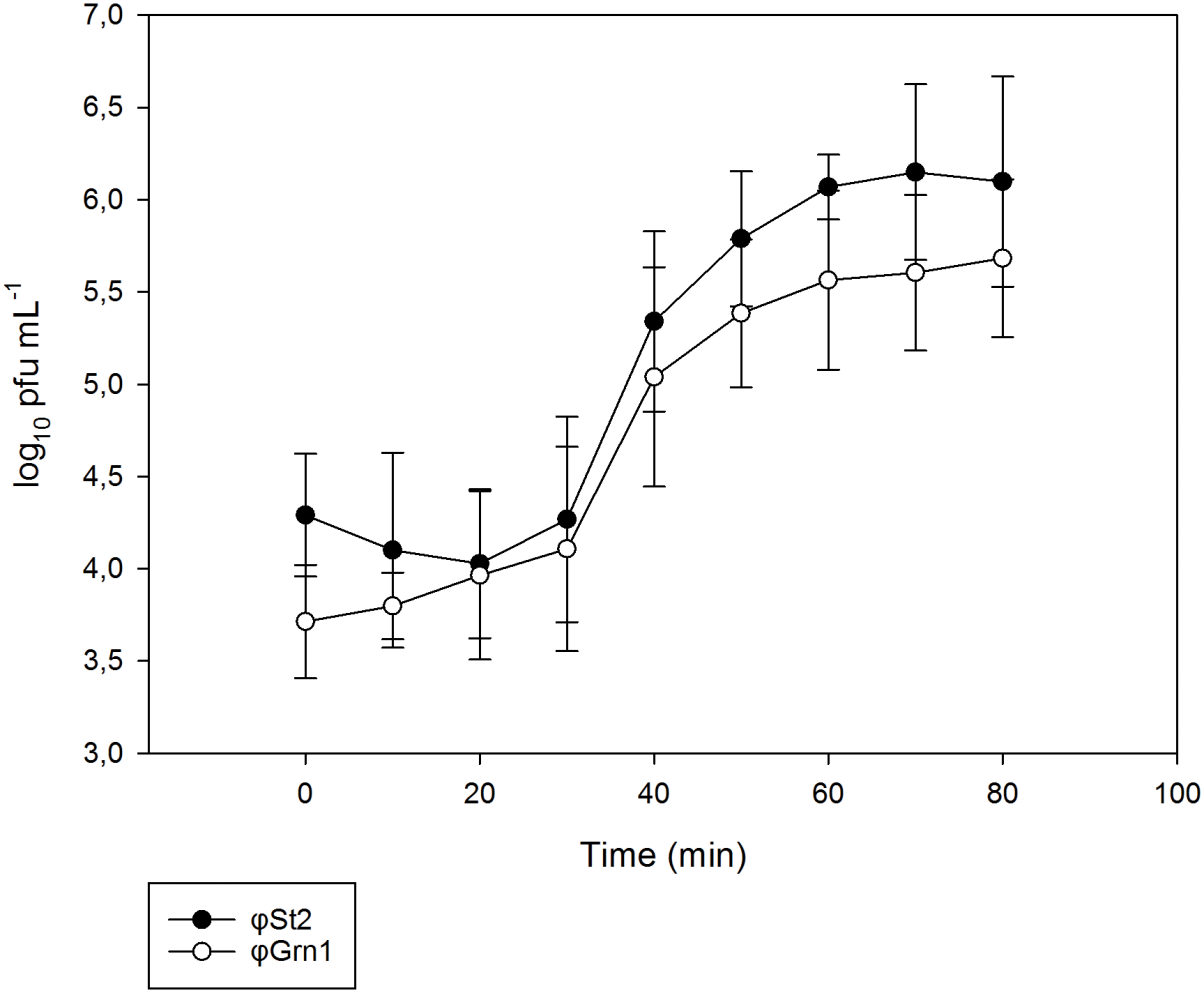
One step growth curve for bacteriophages *φ*St2, latency time: 30 min, burst size: 97 phages per cell and *φ*Grn1, latency time: 30 min and burst size: 44 phages per cell. All values are means ± standard deviation of three independent experiments.

The replication parameters of these phages were determined by one step growth curves showed in Fig 3. Both *φ*St2 and *φ*Grn1 had an approximate latency time of 30 min. However, their burst sizes were quite different since in the case of *φ*St2 it was 97 pfu / cell whereas in the case of *φ*Grn1 the burst size was 44 pfu / cell.

### *In vitro* lytic effect on *Vibrio* strains

**Fig 4:**
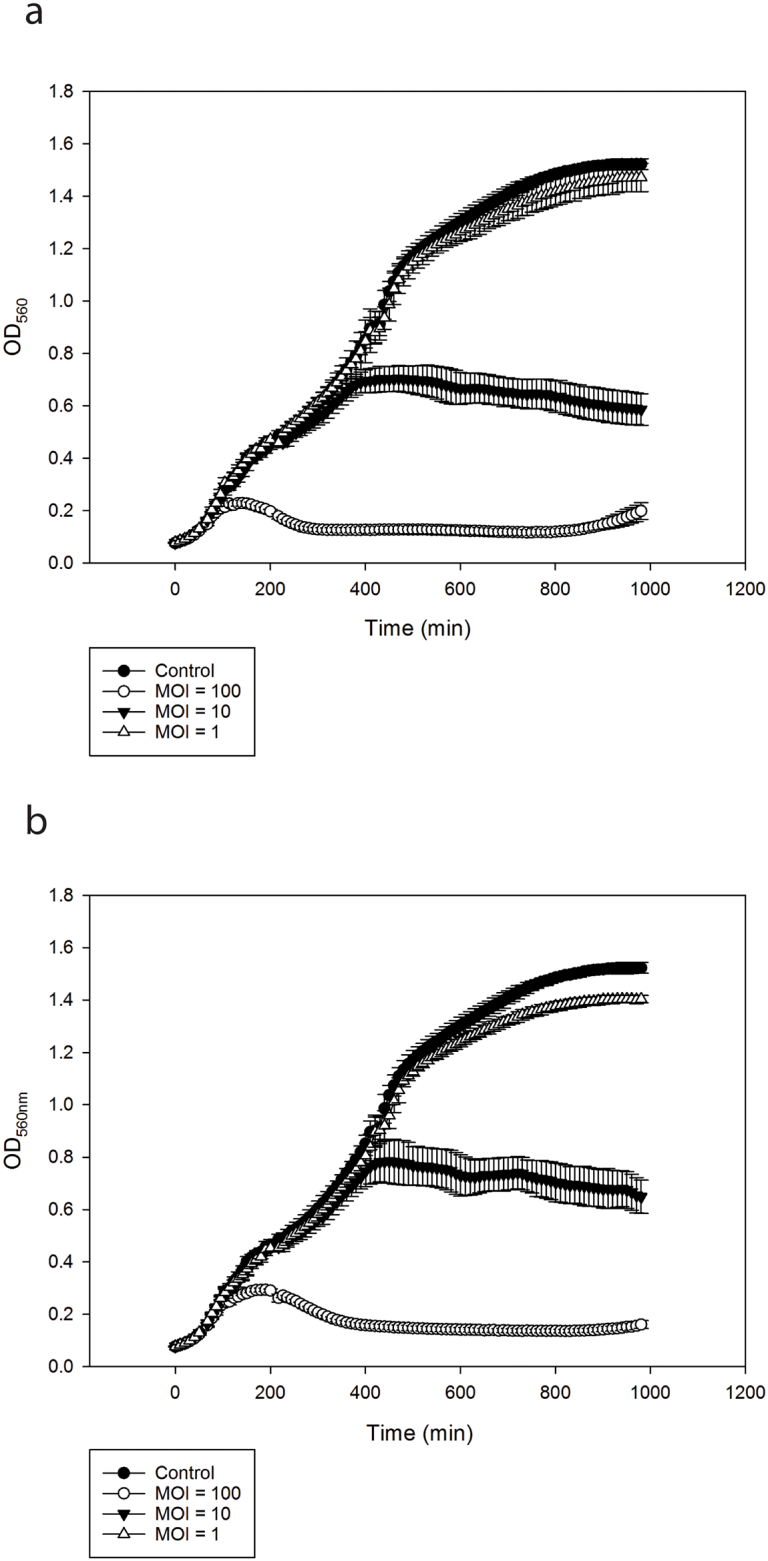
*In vitro* cell lysis experiment of phage (a) *φ*St2 and (b) *φ*Grn1 vs host strain *V*. *alginolyticus* V1. The conditions tested are: control (V1 strain without any phage addition) and 3 different MOI rates used for infecting V1 (MOI = 1, MOI = 10, MOI = 100). The values are means ± standard deviation of the six replicates.

The lytic effect on the host bacteria was tested by infecting fresh cultures of *V*. *alginolyticus* strain V1 at the early exponential phase (OD_560_ ~ 0.15) with *φ*St2 and *φ*Grn1 separately. Lysis was proportional to the MOI used with the lowest (MOI = 1) resulting to no effect and the highest (MOI = 100) to almost complete inhibition of bacteria growth for the whole duration of the experiment (1000 min). The response of the host was similar for both phages with high reproducibility of the results among the replicates (Fig 4a and 4b).

**Fig 5:**
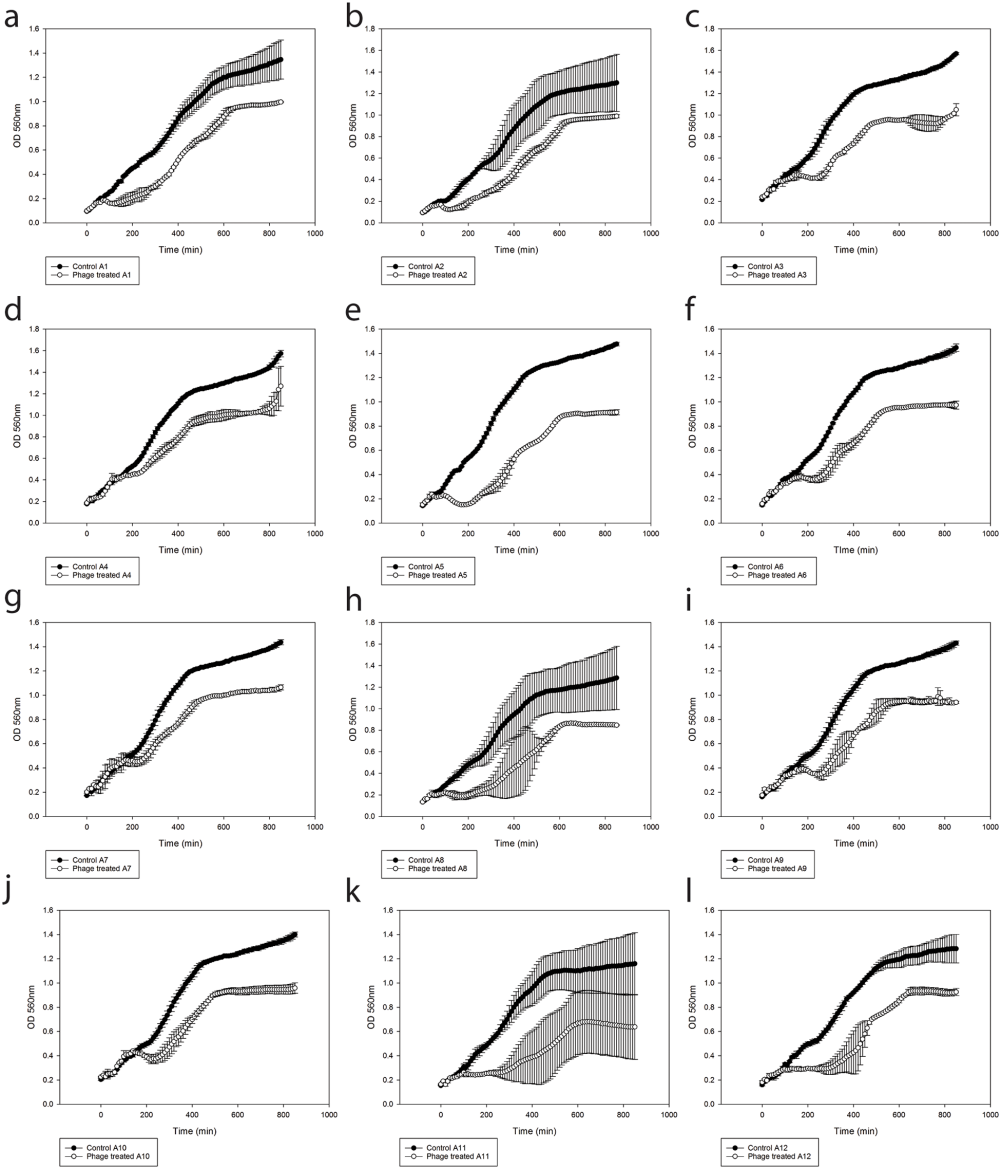
(a) A1, (b) A2, (c) A3, (d) A4, € A5, (f) A6, (g) A7, (h) A8, (i) A9, (j) A10, (k) A11 and (l) A12. *In vitro* cell lysis experiment of phage mixture (*φ*St2 and *φ*Grn1) against 12 presumptive *Vibrio* strains (A1–A12) isolated from the live feed culture, *A*. *salina*. The values are means ± standard deviation of the three replicates.

The phages were tested *in vitro* against twelve bacterial isolates grown in TCBS originated from live feed *A*. *salina* culture. A phage mixture of *φ*St2 and *φ*Grn1 at MOI = 100 significantly affected the growth of all twelve bacterial strains tested (Fig 5a–5l). In all cases, there was a delay in the exponential phase and even when the cultures reached a plateau of growth, the density of phage—treated bacteria was lower compared to their corresponding controls.

### *In vivo* efficacy of phages to control *Vibrio* load in *A*. *salina* culture

**Fig 6:**
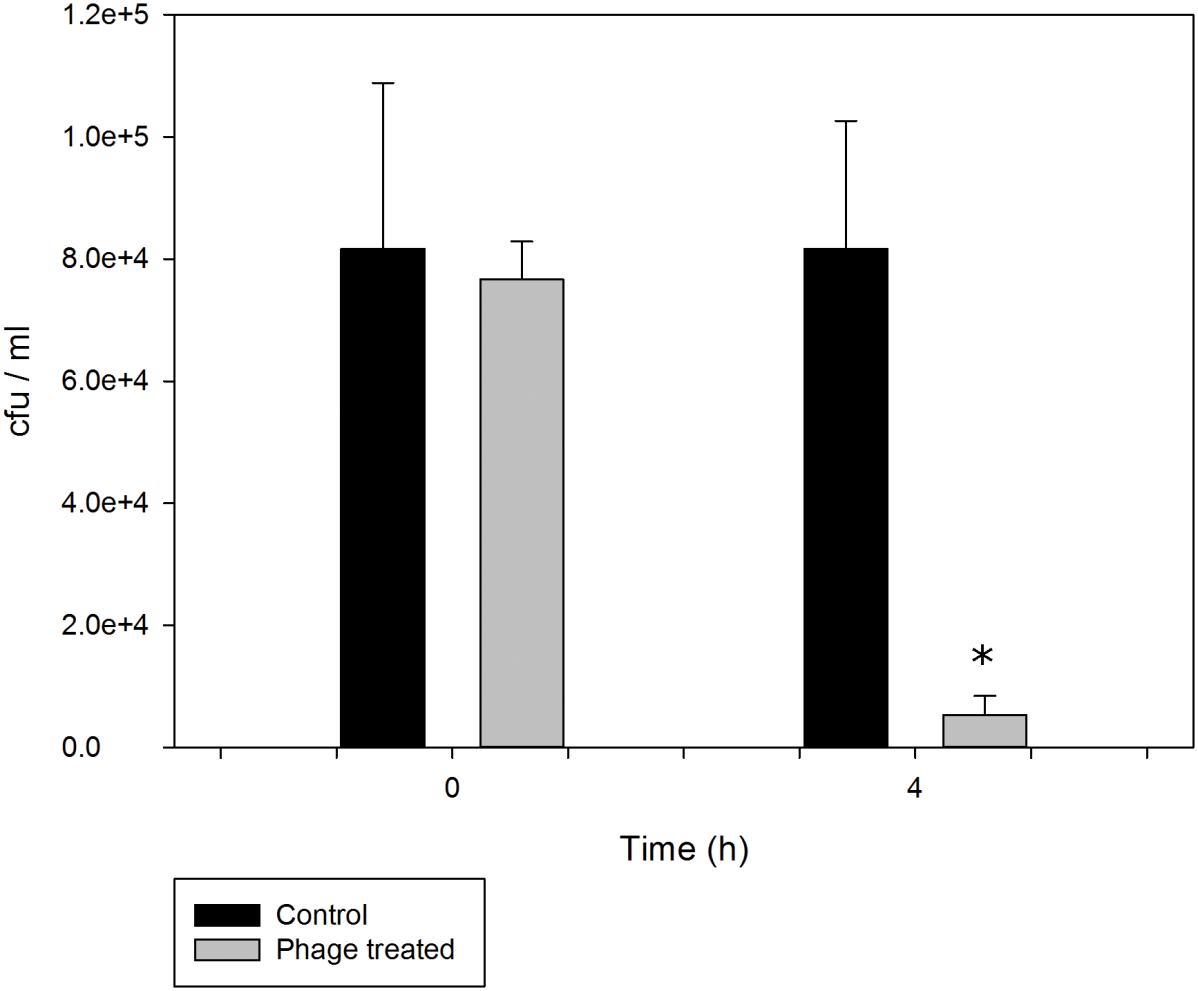
*In vivo* efficacy levels of phages *φ*St2 and *φ*Grn1 administered directly in live feed cultures of *A*. *salina*. The values are means ± standard deviation of triplicates experiment (p < 0.05).

The effect of the phage mixture (*φ*St2 and *φ*Grn1 at MOI = 100) was *in vivo* examined by direct administration in live prey cultures, *A*. *salina*. At t_0_ = 0, the presumptive *Vibrio* count in TCBS plates was 8.1 x 10^4^± 2.7 x 10^4^ cfu mL^-1^ for the control and 7.6 x 10^4^± 0.6 x 10^4^ cfu mL^-1^ for the phage-treated cultures with no statistically significant difference (p: 0.782). After 4 h of incubation, presumptive *Vibrio* count did not change in the control cultures while it became significantly lower in the phage treated ones (p < 0.05). Total *Vibrio* load in phage treated live prey was 5.3 x 10^3^±3.1 x 10^3^ cfu mL^-1^, which is approximately 1.3 orders of magnitude or 93% decrease of the initial total presumptive cultivable *Vibrio* load (Fig 6).

## Discussion

Live feed organisms like *Artemia* are able to bio-accumulate bacteria from the water column [57] acting as a vehicle for pathogen transfer into the hatchery facilities. Bacteriophage therapy in such fragile systems can be a reasonable alternative for the control of microbial diseases and for the prevention of multi—drug resistant bacteria spreading in aquaculture [58]. Since antibiotic resistance mechanisms are irrelevant to mechanisms of phage infection, phages could be successfully employed even against antibiotic—resistant pathogens [59].

In the current study, two novel bacteriophages *φ*St2 and *φ*Grn1 were isolated and characterized. Both phages produced plaques with identical morphology in host bacterial lawn. Observation under TEM resulted in one common virion morphology, similar to the *V*. *alginolyticus* phage *φ*pp2 [60]; all three phages belong to Myoviridae family, but the sizes of *φ*St2 and *φ*Grn1 virions were much larger than *φ*pp2. Bacteriophage *φ*St2 is larger than *φ*Grn1 in both head length (151 nm vs 138 nm) and head width (81 nm vs 74 nm). However, both phages had the same tail length and diameter. They are different from other *V*. *alginolyticus* phages described in literature like *φ*A318 and VAP11 which are classified as Podoviridae viruses with diameter of head 50–55 nm and 60 nm, respectively [61,62]. The head—associated filamentous appendage that was present on all virions’ heads under TEM, is not a common morphological feature of bacteriophages. However, similar devices have been recorded in *Caulobacter crescentus* phages acting as tool for the initial attachment of the phages to the bacterial flagella of the host [63]. Whether this is also its function in the case of *φ*St2 and *φ*Grn1 needs further investigation.

Host range results were identical for *φ*St2 and *φ*Grn1 suggesting that they recognize identical or very similar structures as receptors in their host. Lytic activity of the phages was not limited to the host strain V1, since they were able to infect all 8 *V*. *alginolyticus* strains tested, as well as bacterial strains that belong to *V*. *harveyi* and *V*. *parahaemolyticus* species. In terms of EOP, *φ*St2 and *φ*Grn1 were almost equally efficient against all phage—sensitive bacterial strains, producing plaques within one order of magnitude. According to host range and EOP results, bacteriophages *φ*St2 and *φ*Grn1 are broad host range phages, being able to infect in total 10 out of the 25 strains (40%) tested. However, it should be mentioned that only one strain for *V*. *owensii* and one strain of *V*. *parahaemolyticus* were examined against *φ*St2 and *φ*Grn1, thus, their lytic potential against these two harveyi clade bacterial species needs to be further explored. The conservative nature of the structure of LPS phage receptors on the outer membrane of many of Gram negative bacterial species, could be a possible explanation for this broad lytic spectrum [64]. Further, genetic similarity of these bacterial species which belong to the harveyi clade may also have contributed to this observation [65,66]. The best candidates for phage therapy should be viruses able to lyse the majority of the bacterial target’s strains [67–69]. Thus, *φ*St2 and *φ*Grn1 are suggested as potential phage therapy candidates against infections caused by *V*. *alginolyticus*, since their lytic spectrum include all the *V*. *alginolyticus* strains tested and even strains from other species. Moreover, these phages were able to affect the growth of several strains isolated from an *Artemia* live feed system. It is known that several marine phages can infect different strains of the same species or different closely related species [53,70], however, specificity of phage-host interactions in marine waters is not fully elucidated and needs further investigation [71]. Another similar case of broad host range *Vibrio*—phage is *φ*A318, isolated using *V*. *alginolyticus* host strain, able to infect several *V*. *harveyi* strains [61]. Interestingly, *φ*St2 and *φ*Grn1 had strong lytic effect also against *V*. *alginolyticus* type strain DSM 2171, isolated in Japan from a spoiled horse mackerel responsible for food poisoning [72] expanding therefore their potential applications beyond aquaculture.

RFLP analysis showed that *φ*St2 and *φ*Grn1 are two distinct phages. Three of the restriction enzymes used could not digest the genome of these phages. Bacteriophages refractory to restriction enzyme activity could be explained by several reasons. Among the most prevalent is the natural loss of restriction sites during evolution [73] but also the integration of unusual bases in the viral DNA [74,75]. More importantly, the expression of specific methyltransferase genes that may be contained in the viral genome will cause epigenetic modifications on the viral DNA affecting the restriction sites that endonucleases can recognize [76]. Comparative genomic analysis of these phages would provide further insights to their genetic identity.

One step growth curves defined the biological parameters of *φ*St2 and *φ*Grn1 replicative cycle (latency time and burst size). Bacteriophages *φ*St2 and *φ*Grn1 have similar and relatively short latency time (in both cases about 30 min) compared to other phages [61,77], but differ significantly in their burst sizes (*φ*St2, 97 phages per cell and *φ*Grn1, 44 phages per cell). Phages with short latency period and high burst size are the most appropriate candidates for phage therapy, however, usually high burst sizes are followed by a more extensive latency period [78]. Thus, *φ*St2 and *φ*Grn1 can be considered as good candidates for phage therapy applications since they have relatively short latent period and their burst size seems satisfactory apart from their wide host range.

*In vitro* efficacy of *φ*St2 and *φ*Grn1 showed that both phages were notably effective against their bacterial host *V*. *alginolyticus* strain V1, at MOI = 10 and MOI = 100. On the contrary, at MOI = 1 the effect of phages in the bacterial growth was minimum. According to the literature high MOI rates, usually MOI = 100, are mostly used when phage therapy applied [44,79]. In our case, when MOI = 100 was applied *in vitro* against V1, the bacterium was almost completely eliminated after 100 min and did not recover until the end of the experiment (1000 min). Both *φ*St2 and *φ*Grn1 exhibited a very similar but intense bactericidal activity. The use of phage combination is proposed in order to avoid bacterial resistance against phage infection [70], and there are several reports suggesting the use of phage cocktails to control vibriosis [43,44,69].

Prior to the *in vivo* phage administration, a selection of 12 presumptive *Vibrio* isolates from the *A*. *salina* culture were tested *in vitro* against the combined lytic activity of *φ*St2 and *φ*Grn1. All isolates showed a variable degree of sensitivity to the cocktail of *φ*St2 and *φ*Grn1. Although the strains were not identified to species level it was presumed that they were vibrios based on their efficacy to grow on the Vibrio selective medium but also on colony morphology. These strains were possibly closely related to *V*. *alginolyticus* since as stated earlier according to the literature, this species is considered to be the most prevalent bacterial component in *A*. *salina* cultures [13,31,32].

*In vivo* phage therapy application resulted in significant decrease of the presumptive *Vibrio* load in phage treated live preys. The initial presumptive *Vibrio* load in phage treated *Artemia* was decreased by 93% meaning that 93% less *Vibrio* will enter the hatchery system. Therefore, a treatment with phages in the live feeds prior to their introduction in the hatchery system could effectively reduce the *Vibrio* load in the larval rearing tanks. Controlling and reducing vibrios in fish and invertebrates hatcheries is critical for the final survival and quality of the produced larvae since the possibility for disease outbreak increases proportionally with the increase of the pathogen load. Our experiment did not show a total elimination of *Vibrio* in the live feed. Nevertheless, the reduction but not the complete eradication of the vibrios may also be beneficial since at a later stage, the maturing fish immune system will be exposed to low levels of bacteria helping the fish to develop immunity before their transfer to the open sea. From a practical point of view there are unique advantages in the use of phages as a preventive measure against vibriosis. Firstly, phage treatment procedure requires a relatively short period of time (4 h in our case), which offers the opportunity of disinfecting the live prey exactly before its administration to the larvae in the hatchery. The latter is that *Artemia* is a batch culture and as such, bacterial resistance development against phage infection is rather difficult. A phage treatment protocol could be established on a regular basis for each growth cycle of *Artemia* and phages should be added to the live prey 4 h before administration to the fish. Besides, the use of phages against vibrios would specifically reduce the load of potentially pathogenic bacteria, leaving other species unaltered. Numerous studies using bacteriophages as treatment agents, verify that applications in many aquaculture systems have been successful against many pathogenic bacteria such as *V*. *harveyi*, *V*. *parahaemolyticus* and *V*. *anguillarum* [43,46,48,58,68].

The apparent lytic nature of the phages (also confirmed by the initial genomic analysis not presented here) is an advantage but also a prerequisite for safe phage therapy development since proliferation of resistant bacteria is considered one of the main drawbacks in the use of bacteriophages to control bacteria [80]. It is commonly accepted that bacteriophages are a key factor to determine the bacterial populations in the environment [81,82]. Drawbacks and issues of concern are the eventual resistance development from bacteria against phage infection and the possibility of prophage induction, mutations and horizontal gene transfer [59]. These are events occurring naturally in the environment which however may be augmented and accelerated with careless use of phages, however variation in phage cocktails has the potential to delay or even inhibit the emergence of phage resistance [83,84].

Currently, it is commonly accepted that the need of an innovative and environmentally friendly alternative to antibiotics has become more than necessary. Presence of bacteriophages in environments where pathogenic bacteria occur should be seen as an opportunity that may lead to the development of a successful innovative and environmentally friendly solution. However, phage therapy should always be applied with great caution. We hope that further knowledge of phage—bacterium interactions will contribute to our ability to handle our own microbiota, when bacteriophages are used as biocontrol agents.

## Conclusions

The newly isolated bacteriophages, *φ*St2 and *φ*Grn1, are characterized by broad host range and compelling biological attributes making them potential candidates for phage therapy application. The positive results obtained by *in vitro* and *in vivo* trials, advocate to the fact that phage therapy in aquaculture can be an alternative to antibiotics. Thus, *φ*St2 and *φ*Grn1 can effectively be used in the biological control of the *Vibrio* load in marine hatcheries, through of live prey disinfection.

Next step will be the analysis of the full genome sequencing of *φ*St2 and *φ*Grn1 as well as the further evaluation of the novel disinfection technique on the survival of fish larvae in a controlled experiment.
